# Hemophagocytic Lymphohistiocytosis Associated With Human Herpesvirus-6 (HHV-6) Infection in an Immunocompetent Adult: A Case Report

**DOI:** 10.7759/cureus.54299

**Published:** 2024-02-16

**Authors:** Ateeb Ur Rahman, Fahad Baig, Umar Iqbal Javid Chaudhary, Muhammad Bilal Ashraf, Muhammad Daim Jawaid, Amna Chaudary, Munim Tariq

**Affiliations:** 1 Department of Internal Medicine, Penn State Health, Camp Hill, USA; 2 Department of Medicine, Rashid Latif Medical College, Lahore, PAK; 3 Department of Medicine, Arif Memorial Teaching Hospital, Rashid Latif Medical College, Lahore, PAK; 4 Department of Community Medicine, Rashid Latif Medical College, Lahore, PAK; 5 Department of Internal Medicine, Penn State Health, Camp Hill, PAK; 6 Department of Internal Medicine, The Rotherham NHS Foundation Trust, Sheffield, GBR

**Keywords:** hyperferritinemia, cytokine release storm, human herpes virus type 6, secondary hemophagocytic lymphohistiocytosis (hlh), hemophagocytic lymphohistiocytosis (hlh)

## Abstract

Hemophagocytic lymphohistiocytosis (HLH) is a rare disorder characterized by extreme immune activation and excessive inflammation. It has been reported in patients with familial cases, immunodeficiencies, malignancies, stem cell transplants, and viral etiologies. This report describes acquired HLH associated with Human herpesvirus-6 (HHV-6) infection in a 76-year-old previously healthy male. The patient was admitted to the hospital due to fever, chills, and abdominal pain. The diagnostic workup revealed gallbladder wall thickening on imaging, concerning for cholecystitis. The patient was started on treatment for sepsis. Further clinical deterioration led to an extensive infectious workup. The patient was found to have elevated soluble IL-2Ra levels, and a bone marrow biopsy was performed, which revealed HLH. A positive HHV-6 polymerase chain reaction in the cerebrospinal fluid and serum confirmed the viral infection. Treatment involved the initiation of high-dose steroids, etoposide, and ganciclovir. Despite these interventions, the patient's clinical status worsened, leading to the implementation of comfort measures, and the patient eventually died. This case underscores the importance of considering HHV-6 as a potential cause of HLH in immunocompetent adults. From this case, we infer that a heightened level of vigilance is necessary to recognize and intervene in this challenging condition promptly.

## Introduction

Hemophagocytic lymphohistiocytosis (HLH) is a life-threatening disorder characterized by the upregulation of T-cell activity, including the activation of macrophages, causing a cytokine storm and extensive tissue damage. It involves the endocytosis or ingestion of hematopoietic cells by activated macrophages and is not restricted by the normal limitations of the immune system. HLH can be primary or acquired, and the pattern of T-cell activation can vary depending on the cause [1]. While HLH is predominantly identified in pediatric patients, this condition can be diagnosed across all age groups [2]. HLH has been reported in association with malignancies, most commonly lymphoid cancers and leukemias, but also with solid tumors, solid organ and hematopoietic cell transplants, children with inherited immunodeficiency disorders [3], and viral infections [4]. Epstein-Barr Virus (EBV), cytomegalovirus (CMV), and HIV are the most common viral infections associated with HLH [5]. Diagnosis is difficult due to the significant overlap between clinical manifestations and the rarity of this syndrome. HLH can present as lymphadenopathy, hepatosplenomegaly, jaundice, or fever. Here, we report an unusual case of acquired HLH associated with human herpesvirus-6 (HHV-6) in an immunocompetent elderly male patient.

## Case presentation

A 76-year-old male with no known medical history presented to the ER with a high-grade fever, rigors/chills, and intermittent confusion for the past few days. A review of the systems revealed generalized abdominal pain and mild shortness of breath.

Upon presentation, the patient's initial vital signs were blood pressure (BP) of 110/55 mmHg, heart rate (HR) of 92 beats per minute, respiratory rate (RR) of 22 breaths per minute, and a temperature of 102°F. Physical examination showed mild bibasilar crackles; the abdomen was soft, mildly distended, and diffusely tender, without peritoneal signs. +1 bilateral pitting edema was observed in the lower extremities. The patient had normal motor strength and sensation in both upper and lower extremities, and cranial nerves II through XII function was intact.

Laboratory work on admission showed an elevated white cell count of 15,000/uL, aspartate aminotransferase (AST) of 65 U/L, alanine aminotransferase (ALT) of 70 U/L, and creatinine of 1.8 mg/dL. The patient was started on treatment for sepsis of unknown etiology with broad-spectrum antibiotics, including vancomycin, cefepime, and metronidazole. Initial imaging findings on gallbladder ultrasound indicated acute cholecystitis, but a hepatobiliary iminodiacetic acid (HIDA) scan revealed only gallbladder wall thickening without pericholecystic fluid or stones. General surgery and gastroenterology teams were consulted to evaluate the patient, but they did not feel that the patient would benefit from cholecystectomy given the inconclusive findings on imaging studies.

**Figure 1:**
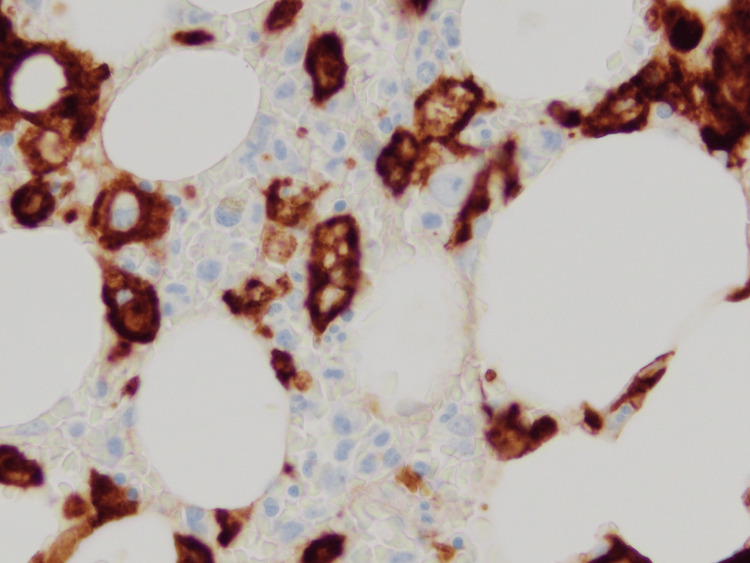
Immunohistochemical staining for CD163 on the bone marrow biopsy. CD: Cluster of differentiation. CD163 is a marker of cells from monocyte/macrophage lineage. Its upregulation can facilitate phagocytosis.

**Figure 2:**
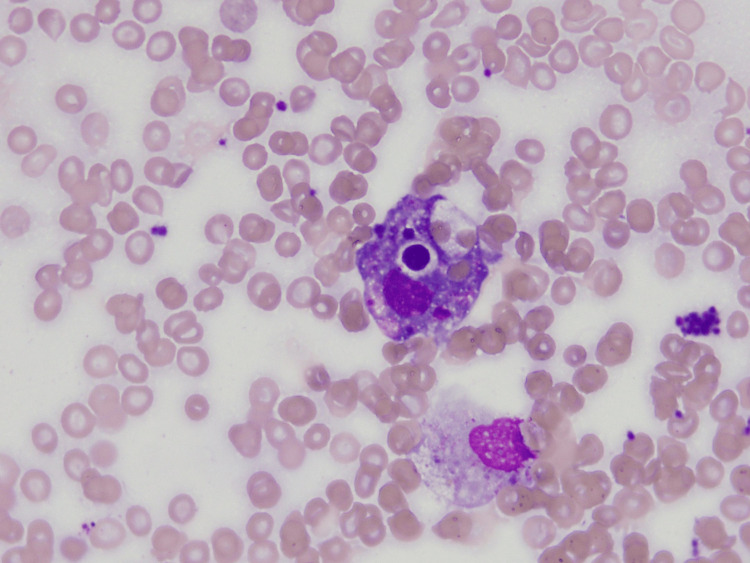
Hemophagocytosis on a Wright-Giemsa-stained aspirate.

Despite antibiotics, the patient experienced daily high-grade fevers (up to 104°F) and worsening renal function, with creatinine increasing to 2.6 mg/dL, AST to 300 U/L, and ALT to 350 U/L. The patient became hemodynamically unstable, with mean arterial pressures (MAPs) dropping to 50 mmHg, and was transferred to the intensive care unit for further monitoring. Intravenous vasopressors were started for blood pressure support. Elevated ferritin levels up to 7,500 ug/L prompted a hematology/oncology consultation for suspected HLH. A bone marrow biopsy was conducted and the pathology was suggestive of hemophagocytosis (Figures 1-2).

Soluble IL-2 receptor alpha (CD25) levels were significantly elevated, reaching 65,475 pg/mL (normal range: 532-1,891 pg/mL). High-dose steroids (dexamethasone 1 mg/kg) and etoposide therapy were initiated. An extensive infectious and autoimmune workup was conducted to identify the cause of secondary HLH. A lumbar puncture showed positive CSF for HHV-6 PCR. Ganciclovir treatment was immediately started. Given his deteriorating condition and worsening multiorgan failure, the family opted for comfort care measures, and the patient subsequently passed away.

## Discussion

HLH is an immune deficiency disorder that is characterized by immune activation and widespread organ damage. There are two types: primary/familial and secondary. Both types of HLH are life-threatening. Due to its rarity, it can easily go unnoticed in healthcare settings. The most common symptoms include persistent fever, rashes, seizures, low appetite, and fatigue.

**Figure 3:**
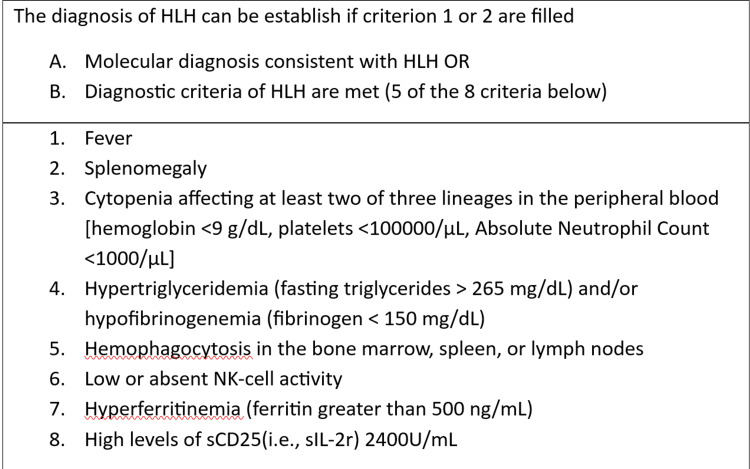
HLH-2004 diagnostic criteria. HLH: Hemophagocytic lymphohistiocytosis.

The HLH -2004 guidelines include the five criteria from the 1991 guidelines, with three additional criteria added, which are listed in Figure 3.

For a diagnosis, five of the eight established criteria must be met [6]. However, it is important to note that patients with a molecular diagnosis indicative of HLH may not necessarily meet all the diagnostic criteria. HLH should always be considered as a differential diagnosis for hyperferritinemia [7]. In adults, ferritin values greater than 7,000-10,000 µg/L, and rarely, over 100,000 µg/L, are characteristic of HLH. In children, ferritin levels greater than 10,000 µg/L are more than 90% sensitive and specific for HLH; however, additional criteria must be met to make the diagnosis. Although hyperferritinemia is not a precise indicator of HLH in adults, ferritin levels within this range strongly suggest its presence. Therefore, a thorough evaluation of the numerous clinical features is required to confirm the diagnosis of HLH in adult patients [8][9]. The HLH-2004 criteria, created for children, have not been formally validated for adults and are still based on professional judgment. The modified HLH-2004 criteria have been used in several case studies [6,10-12].

The HScore, a new online calculator for diagnosing HLH, demonstrates diagnostic sensitivity and specificity of 100% and 80% for children, and 90% and 79% for adults, respectively (Table 1). According to a multicenter cohort study, as the patient's clinical status deteriorates, the performance in adults is comparable between the modified HLH-2004 guidelines and the HScore. While maintaining the same sensitivity, the specificity drops to 73% (Table 2) [12,13]. It can be found online at https://www.mdcalc.com/calc/10089/hscore-reactive-hemophagocytic-syndrome and https://saintantoine.aphp.fr/score/.

**Table 1:** HScore (HLH probability calculator). *HIV-positive or receiving long-term immunosuppressive therapy (i.e., glucocorticoids, cyclosporine A, azathioprine). †Defined as a hemoglobin level of 9.2 g/L and/or a leukocyte count ≤5 × 109/L and/or a platelet count ≤110 × 109/L. HLH: Hemophagocytic lymphohistiocytosis.

Parameter (criteria for scoring)	No. of points
Known underlying immunosuppression*	0 (no)
	18 (yes)
Temperature (°C)	0 (<38.4 C)
	33 (38.4–39.4 C)
	49 (>39.4 C)
Organomegaly	0 (no)
	23 (hepatomegaly or splenomegaly)
	38 (hepatomegaly and splenomegaly)
No. of cytopenias†	0 (1 lineage)
	24 (2 lineages)
	34 (3 lineages)
Ferritin (μg/L)	0 (<2000)
	35 (2000-6000)
	50 (>6000)
Triglyceride (mmol/L)	0 (<1.5)
	44 (1.5-4)
	64 (>4)
Fibrinogen (g/L)	0 (>2.5)
	30 (≤2.5)
Aspartate aminotransferase (U/L)	0 (<30)
	19 (≥ 30)
Hemophagocytosis on bone marrow aspirate	0 (no)
	35 (yes)

**Table 2:** HScore. Note: The best cut-off value for the HScore was 169, corresponding to a sensitivity of 93%, specificity of 86%, and accurate classification of 90% of the patients [12].

HScore	Probability of hemophagocytic syndrome
≤90	<1%
91-100	~1%
101-110	1-3%
111-120	3-5%
121-130	5-9%
131-140	9-16%
141-150	16-25%
151-160	25-40%
161-170	40-54%
171-180	54-70%
181-190	70-80%
191-200	80-88%
201-210	88-93%
211-220	93-96%
221-230	96-98%
231-240	98-99%
≥241	>99%

This case underscores the challenges in diagnosing secondary HLH, particularly in older patients with a variable clinical presentation. The discovery of HHV-6 infection as the underlying cause highlights the importance of considering viral etiologies in secondary HLH, though there are multiple documented instances of HLH in children resulting from HHV-6 infection [14]. In the absence of therapeutic intervention, the overall mortality rate of patients with HLH is 40% [15]. A comprehensive study involving 162 adult patients with HLH showed that 94 (58%) survived; among those who did not survive, roughly half succumbed within one month of diagnosis, particularly those with hematologic malignancies [16]. In adults, the prognosis is worse for those with underlying malignancies, older age, and certain markers of disease severity [17]. To target the hyperinflammatory response, treatment is initiated with high-dose steroids [18,19] and etoposide [15], with studies showing full remission after a steroid course [18,19]. HHV-6 has a preference for lymphatic tissues and exhibits growth within peripheral blood mononuclear cells (PBMCs) [20].

## Conclusions

Our case report highlights that secondary HLH can occur due to viral infection in an immunocompetent individual. Early detection and immediate treatment are essential for achieving better results. HLH has an extremely high mortality rate due to its complicated presentation and late diagnosis. Further research, especially with regard to treatment modalities, such as immunomodulatory drugs and cytokine-targeted therapies, is important to curb this life-threatening disease.
